# The Impact of Bacillus Calmette–Guérin Vaccination and *Mycobacterium bovis* Infection on Diagnostic Antibody Tests for Mycobacterial Infections

**DOI:** 10.3390/vaccines13060578

**Published:** 2025-05-28

**Authors:** Thomas Holder, Nick Robinson, Gareth J. Jones

**Affiliations:** 1 TB Immunology and Vaccinology, Bacteriology Department, Animal and Plant Health Agency, Addlestone KT15 3NB, UK; 2 Animal and Plant Health Agency, Lab Services Group, Starcross EX6 8PE, UK

**Keywords:** bovine TB, bTB, BCG, MAP, serology, IDEXX, Enferplex

## Abstract

Background: Bovine tuberculosis (bTB) is an infectious disease which causes significant damage to the farming industry and remains a disease of global significance. Although control strategies have focused on a test and cull approach primarily based around specific cell-mediated immune responses, serological assays are increasingly being used as a supplementary test alongside skin testing and interferon-gamma release (IGRA) assays. The UK is moving towards the use of the Bacillus Calmette–Guérin (BCG) vaccination of cattle as an additional targeted control tool against bTB. However, there are concerns over its potential impact on the outcomes of bTB diagnostic tests and other non-TB assays, such as serological tests for *Mycobacterium avium* subsp. *paratuberculosis* (MAP). Methods: We investigated the performance of commercially available serology tests designed to detect bTB and MAP using serum samples from BCG-vaccinated animals which were subsequently infected with *Mycobacterium bovis* (*M. bovis*). Results: BCG vaccination per se did not significantly impact the specificity of serological diagnostic tests for bTB or Johne’s disease. However, increased numbers of false-positive responses in bTB serology tests were seen in BCG-vaccinated animals 3 weeks following a tuberculin skin test, where up to 23% and 54% of animals gave a positive result in IDEXX and Enferplex tests, respectively. Furthermore, *M. bovis* infection gave rise to false-positive test results for Johne’s disease, irrespective of the animals’ prior BCG vaccination status. Conclusions: Caution should be taken when assessing results from serology tests for bTB if tuberculin skin testing has occurred shortly before collection of blood from BCG-vaccinated cattle. Furthermore, these results highlight the potential for misdiagnosis of MAP infection when using serology tests in bTB-infected cattle.

## 1. Introduction

Bovine tuberculosis (bTB) is a chronic disease caused by infection with members of the *Mycobacterium tuberculosis* complex (MTBC) and is observed in numerous domestic and wildlife mammals, including cattle, buffalo, goats, deer, camelids, possums, badgers, and wild boar [1]. In many countries, including the UK, bTB in cattle is more often due to infection with *Mycobacterium bovis* (*M. bovis*) rather than other members of the complex (e.g., *M. caprae* or *M. tuberculosis*). The disease in cattle is a global issue, with a recent meta-analysis of the published literature estimating prevalence rates of 10.3%, 13.8%, 17.8%, 33.6%, and 20.5% in Africa, Asia, Europe, North America, and South America, respectively (although estimates may be influenced by a lack of reported data from low- and middle-income countries) [2]. Furthermore, bTB is believed to be responsible for a global economic loss of USD 3 billion a year [3] and poses a significant public health threat due to its potential for zoonotic transmission, particularly in developing countries around the world [4,5,6]. Currently, Great Britain (GB) employs a test-and-slaughter control policy, which is based on the application of diagnostic tests that detect cell-mediated immune (CMI) responses: the single intradermal comparative cervical tuberculin (SICCT) skin test is used as the primary screening test, with the interferon-gamma release assay (IGRA) used as a supplementary test to ensure maximal detection of infected animals. These control strategies are particularly costly in GB, with estimates in England of a cost of GBP 70 million per year to the taxpayer with an additional cost of GBP 50 million paid to farmers for compensation relating to the culling of infected animals [7]. More recently, serological-based assays have also been utilised as an ancillary tool, as they have been shown to detect infected animals that are unresponsive in CMI-based tests [8]. Furthermore, it has been previously shown that the application of a tuberculin skin test boosts humoral immune responses (known as an anamnestic response) and that this can greatly affect the sensitivity of humoral immune response-based assays [9,10,11].

Alongside test and cull measures, cattle vaccination has the potential to offer additional benefits as a supplementary control strategy [12], and the lead candidate vaccine for bTB in GB is Bacillus Calmette–Guérin (BCG) Danish strain 1331. Unfortunately, BCG vaccination sensitises cattle to the current reagents (i.e., purified protein derivatives of tuberculin) used in both skin testing and IGRA diagnostic assays, precluding its previous use alongside test and cull strategies due to the generation of false-positive test results [13]. However, the development of defined antigen reagents capable of detecting infected animals amongst BCG-vaccinated ones (DIVA) [14] has meant that BCG vaccination may now be a viable option for use in bTB control strategies.

Given the potential increased use of serology-based diagnostic tests in bTB control programmes, the impact of BCG vaccination on these tests should also be evaluated. To date, there have been a limited number of studies assessing the impact of BCG vaccination on the diagnosis of TB using serology-based assays. One study in cattle found no responses to defined mycobacterial antigens (ESAT-6, CFP-10, MPB59, MPB64, MPB70, MPB83, Acr1, and PstP-1) in either vaccinated or control animals prior to infection [15]. However, more frequent IgG responses were seen post-infection in the unvaccinated group when compared to the BCG-vaccinated group. Another study in white-tailed deer showed lower levels of detectable circulating antibodies to *M. bovis* in BCG-vaccinated animals post-infection when compared to unvaccinated deer. As with the previous study, no serological responses to specific *M. bovis* antigens were seen prior to infection in either group of animals [16]. In our current study we have utilised the Enferplex TB serology assay. The DIVA potential of an earlier version of this assay has been previously explored. When assessed post-skin testing, no serological responses were seen in cattle vaccinated with BCG. However, the study did not include animals which were vaccinated with BCG and subsequently infected with *M. bovis* [17].

Furthermore, there is a current knowledge gap in the impact of BCG vaccination on diagnostic test outcomes for other mycobacterial infections of cattle. For example, Johne’s disease, caused by infection with *Mycobacterium avium* subsp. *paratuberculosis* (MAP), is a disease of global concern in a wide range of species but particularly in ruminants. The results of a recent survey conducted across 48 countries demonstrated variable prevalence rates, with values between 1 and >40% for herd-level estimates and between 1 and 15% for within-herd estimates, for dairy cattle alone [18]. Although the disease is not notifiable in GB, animals are commonly tested with serological assays (e.g., the IDEXX MAP antibody test) using either serum or milk samples, particularly if displaying clinical signs. Given that this assay detects antibodies against whole mycobacteria cell lysate, there is a concern that BCG vaccination may induce cross-reactive antibody responses. Indeed, false-positive test results in MAP ELISA assays have been observed in cattle infected with *M. bovis* [19,20,21], although this has not been evaluated in non-infected BCG-vaccinated animals.

Thus, to investigate the impact of BCG vaccination and *M. bovis* infection on diagnostic antibody tests for bTB and Johne’s disease, we utilised serum samples collected from a historic BCG vaccination/experimental *M. bovis* infection study.

## 2. Materials and Methods

### 2.1. Animals and Experimental Protocol

The animals and experimental protocol for this study have been described previously [22]. The experimental unit for this study was the individual animal. Briefly, 28 male Danish Holstein or Holstein/cross calves sourced from Denmark (which is an officially bTB-free country) were randomly assigned to either a BCG vaccination group (*n* = 14) or a non-vaccinated control group (*n* = 14). Group sizes were used to mitigate any potential loss of animals during the vaccination phase. Calves were vaccinated at between 45 and 60 days of age via the subcutaneous route with a mean average BCG dose of 0.38 × 10^6^ CFUs (range of 0.2 × 10^6^ CFUs and 0.55 × 10^6^ CFUs; BCG Danish strain 1331; AJVaccines, Copenhagen, Denmark). After a vaccination period of 52 weeks, 12 animals from each group were randomly selected for the infection stage (although sample size calculation based on an alpha error of 5% and power of 95% suggested a minimum sample size of 10 animals per group, 12 per group were selected to mitigate potential animal loss). All 24 animals were experimentally infected with 7600 CFUs of *M. bovis* (UK field strain AF2122/97) via the endobronchial route as previously described [14]. At 13 weeks post-infection, post-mortem examinations were performed and the severity of bTB pathology was assessed, which demonstrated a significant reduction in gross pathology scores in the vaccinated group [22]. Blood samples were taken at regular intervals throughout the study. There were no criteria for excluding samples for analysis, although for some time points it was not possible to sample all animals.

### 2.2. Ethical Approval

The study was performed in accordance with the UK Animals (Scientific Procedures) Act 1986 and associated guidelines (EU Directive 2010/63/EU for animal experiments). Animal procedures and experimental protocols were approved by a named institutional committee (i.e., APHA Animal Welfare and Ethical Review Board; reference PF7D840A5-2-006v2).

### 2.3. Skin Testing

Skin tests using tuberculin reagents (PPD-A; 25,000 IU/mL and PPD-B; 30,000 IU/mL; Thermo Fisher [Prionics], Lelystad, The Netherlands) or a fusion protein consisting of the mycobacterial antigens ESAT-6, CFP-10, and Rv3615c (DST-F; 300 µg/mL; Lionex, Braunschweig, Germany) were performed as previously described [22] and in compliance with the Instructions for Tuberculosis Testing in Bovines (available through the APHA Vet Gateway). Animals were skin tested with tuberculin and DST-F reagents both prior to infection (week 44) and again 12 weeks post-infection (week 64).

### 2.4. Serum Collection

Blood samples were collected at regular timepoints throughout the study, drawn from the jugular vein of all animals. Serum vacutainers were centrifuged (1500× *g*, 15 min), and the serum was decanted into a clean tube and frozen at −20 °C until required for antibody testing.

### 2.5. IDEXX M. Bovis Antibody Test

The IDEXX *M. bovis* antibody test is based upon antibody responses to the immunodominant proteins MPB70 and MPB83, a cocktail of which is immobilised onto the ELISA test plate. The test was performed as per the manufacturer’s instructions (IDEXX Laboratories Ltd., Westbrook, ME, USA). Briefly, serum samples were diluted 1:50 before being transferred to the test plate in duplicate, incubated at room temperature (RT) for 1 h before washing and the addition of conjugate for 30 min, after which plates were washed before the addition of the substrate. Reactions were stopped after a 15 min incubation period at RT and plates were read at 450 nm using a Multiscan FC plate reader (Thermo Scientific, Waltham, MA, USA). Results are presented as an S/P ratio for each individual animal, i.e.,

(sample OD minus mean negative plate control OD)
(mean positive plate control OD minus mean negative plate control OD)

A positive result is an S/P ratio ≥ 0.3. Test performance estimates: specificity of 98% and sensitivity of 64.3% [23].

### 2.6. IDEXX MAP Antibody Test

The test was performed as per the manufacturer’s instructions (IDEXX Laboratories Ltd.). Briefly, samples were diluted 1:20 (serum) using the sample diluent and incubated at RT for 1 h before being transferred to the test plate and further incubated for 45 min at RT before washing and the addition of conjugate. After a 30 min incubation at RT, plates were washed again before the addition of TMB substrate. The stop solution was added after a 10 min incubation period at RT, and the plates were read at 450 nm using a Multiscan FC plate reader. Results are presented as an S/P percentage (%), i.e.,

(sample OD minus mean negative plate control OD) × 100
(mean positive plate control OD minus mean negative plate control OD)

A positive result is a sample with an % S/P value of ≥55%. Test performance estimates: specificity of 99.2% (of 717 MAP-negative cattle) and sensitivity of 64.7% (of 68 faecal culture-positive cattle) (APHA internal validation report, 2020). The test also has a “suspect range” using a % S/P value of between 45 and 55%.

### 2.7. Enferplex TB Antibody Test

The Enferplex multispot ELISA test is based upon antibody responses to 11 antigens spotted onto each well of an ELISA plate: (Rv2975p652; PPDB; Rv2873 [MTB83]; Rv2975; Rv2031-Rv1886c [Ag85]; Rv3875 [ESAT6]-Rv3874 [CFP10]; Rv2626; Rv0251c; and Rv2031c) [24]. The test was performed as per the manufacturer’s instructions (Enfer Scientific, Kildare, Ireland). Briefly, serum samples were diluted 1:200 using sample dilution buffer (Enfer Buffer B, Enfer Scientific) and mixed before being transferred to the test plate. Plates were incubated at 37 °C for 60 min with agitation (900 rpm), then washed 6 times with 1X Wash (Enfer Wash buffer, Enfer Scientific) and aspirated. Sheep anti-bovine IgG-HRP detection antibody (1:20,000 dilution; Bethyl Laboratories, Montgomery, TX, USA) was added, and the plates were incubated at 37 °C for 60 min with agitation (900 rpm). The plates were washed as above before the addition of a chemiluminescent substrate. Relative light units (RLU) were immediately captured (220 s exposure) using a Quansys Biosciences Q-ViewTM LS imager and Q-ViewTM software (v 3.1.15). The results were defined using the Enferplex Bovine TB Macro developed by the manufacturer, based on individual antigen thresholds after subtracting the RLU value obtained from a blank spot. This macro uses different thresholds to analyse the raw data and generate binary test results (i.e., positive or negative) for individual spots in two different situations, one that prioritises sensitivity (noted as Hi Se) and one that prioritises specificity (noted as Hi Sp). An overall positive test result (for either situation) is a sample with ≥2 spots positive. For evaluating changes in spot intensity, responses were displayed as multiples above the cut-off for each individual spot. Test performance estimates: specificity in skin test-boosted cattle 98.8% (Hi Sp); specificity in un-boosted cattle 98.4% (Hi Se) and 99.7% (Hi Sp); and sensitivity in skin test-boosted *M. bovis* culture-confirmed cattle 93.9% (Hi Se and Hi Sp) [24].

### 2.8. Statistical Analysis

Analyses were performed using GraphPad Prism 8.4.2. The normality of the data was assessed using the D’Agostino and Pearson test, and the IDEXX MAP antibody test results were analysed using the Kruskal–Wallis test with Dunn’s multiple comparisons test (compared to week 0 values).

## 3. Results

### 3.1. M. bovis-Specific Antibody Responses

**Figure 1:**
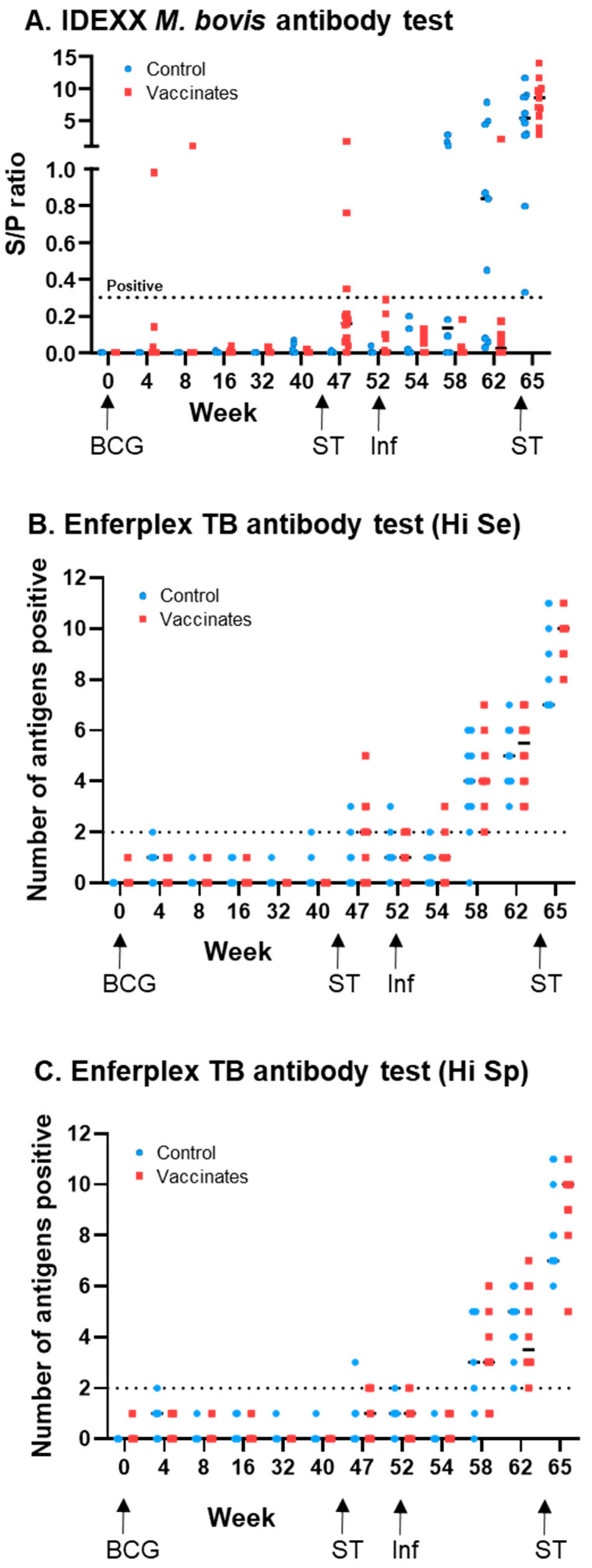
***M. bovis* serology responses following BCG vaccination and *M. bovis* infection using commercially available tests**. Antibody responses to the IDEXX *M. bovis* antibody test (**A**), the Enferplex TB antibody test (high sensitivity interpretation) (**B**), and the Enferplex TB antibody test (high specificity interpretation) (**C**). Each symbol represents an individual animal. Test positive/negative cut-offs are displayed with a dotted line. Unvaccinated control animals are represented in blue and BCG-vaccinated animals in red. Arrows indicate timepoints where animals were vaccinated (BCG), infected with *M. bovis* (Inf), or skin tested (ST).

Figure 1 shows the serum antibody responses using IDEXX (Figure 1A), Enferplex Hi Se (Figure 1B) and Enferplex Hi Sp (Figure 1C) tests. The percentage of test-positive animals within the BCG-vaccinated and unvaccinated cattle is also summarised in Table 1. All animals were test negative (to all tests) at the start of the experiment (w0). For serum samples taken prior to skin testing and *M. bovis* infection (i.e., up to and including w40), there were two IDEXX-positive and two Enferplex (Hi Se)-positive animals. The IDEXX positives were both from the BCG-vaccinated group, while the Enferplex positives were both from the control unvaccinated group. Following a skin test (at w44), there was a transient increase in seropositivity in both the IDEXX and Enferplex tests, with 3 IDEXX-positives at w47 only (all within the BCG group) and 11 Enferplex (Hi Se)-positives between weeks 47 and 52 (8 BCG-vaccinated and 3 unvaccinated controls). Following *M. bovis* infection (at w52), the IDEXX test showed an earlier test positivity in the unvaccinated control group compared to the BCG-vaccinated group, the responses of which only became strongly apparent after a skin test at w64, when all animals then became IDEXX positive. In contrast the Enferplex test post-infection showed test positives increasing at a similar rate within the BCG-vaccinated and unvaccinated control cattle groups. The two Enferplex test interpretations showed broadly similar results, but compared with Enferplex (Hi Se), the Enferplex (Hi Sp) (Figure 1C) test had fewer false positives prior to infection, i.e., 1 (rather than 2) false positive in non-infected animals prior to skin testing, and 8 (rather than 11) false positives in non-infected animals post-skin testing. Post-infection, the kinetics for test positivity were slightly delayed when using the Enferplex (Hi-SP) readout: 6 weeks post-infection (i.e., w58), all but one animal was test positive using the Enferplex (Hi-Se) readout, whereas six animals remained test negative using the Enferplex (Hi-SP) readout. However, by 10 weeks post-infection (i.e., w62), all animals were test positive, irrespective of the readout used.

**Figure 2:**
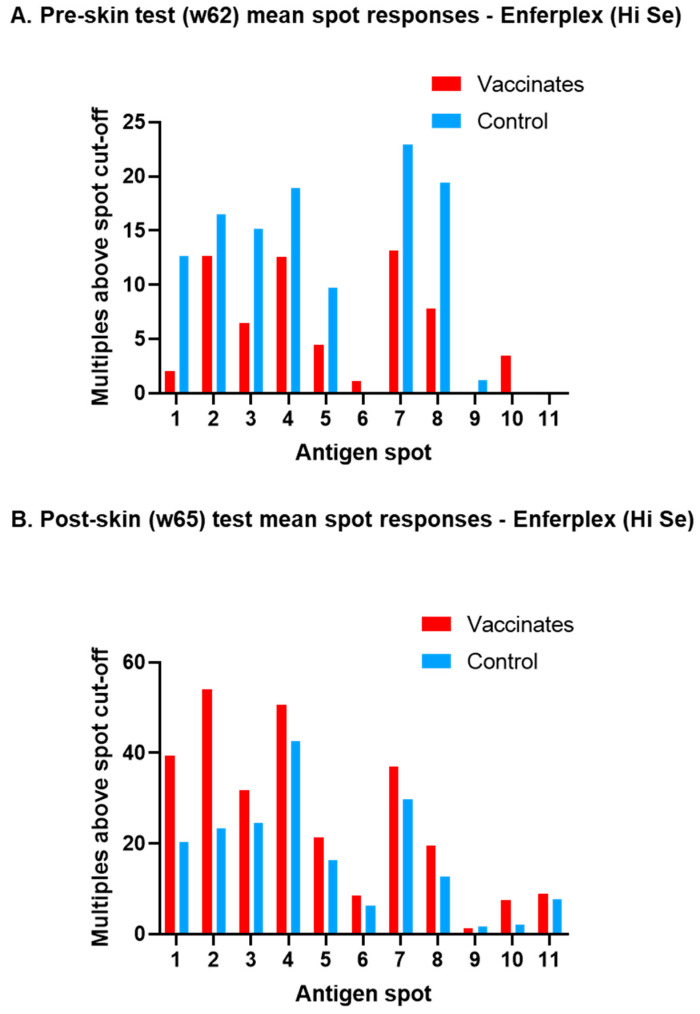
**Mean spot responses on the Enferplex TB antibody test for *M. bovis-*infected animals**. Mean responses to each spot for both the pre-skin test at week 62 (**A**) and the post-skin test at week 65 (**B**) using the high sensitivity cut-off applied. Unvaccinated control animals are represented in blue and BCG-vaccinated animals in red.

Why the Enferplex test showed no difference in antibody responses between BCG-vaccinated and unvaccinated cattle post-infection was investigated. Figure 2 shows for each antigen spot the mean spot response (as multiples above the spot cut-off) of Enferplex (Hi Se)-positive animals pre-skin test (w62, Figure 2A) and post-skin test (w65, Figure 2B). Spots are linked with a line for ease of viewing the two cattle groups. The data show that after infection, but before the skin test (Figure 2A), unvaccinated control cattle have stronger individual spot responses compared to BCG-vaccinated cattle. This switches post-skin test (Figure 2B), where BCG vaccinates have stronger spot responses compared to unvaccinated cattle. Hence, the Enferplex assay can show a similar initial dominance of antibody responses in unvaccinated over BCG-vaccinated cattle post-infection (when samples were taken prior to skin testing), but this is hidden by the nature of the Enferplex test readout (that of “number of spots positive”).

### 3.2. Post-Infection Antibody Versus Interferon-Gamma Test Kinetics

**Figure 3:**
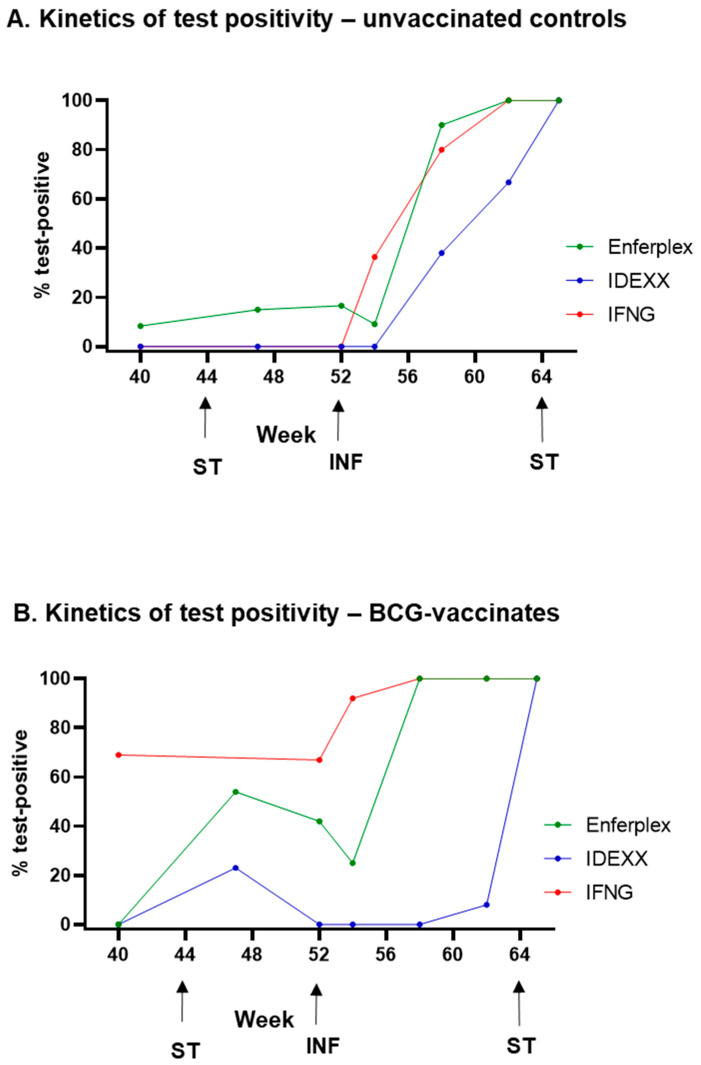
**Comparison of *M. bovis* test outcomes.** Positivity rates for control animals (**A**) and BCG-vaccinated animals (**B**) measured using different *M. bovis*-specific assays. The Enferplex TB antibody test (high sensitivity interpretation) is represented in green, the IDEXX *M. bovis* antibody test is represented in blue, and the Bovigam IGRA is represented in red. Arrows indicate timepoints where animals were skin tested (ST) or infected with *M. bovis* (INF).

The kinetics of antibody test positivity (IDEXX and Enferplex Hi Se) in infected cattle was compared to the interferon-gamma (IFNG) test for these experimental cattle (see [22] for IFNG data). Figure 3 shows the proportion (%) of animals that were test positive pre- and post-*M. bovis* infection (weeks 40 to 65). In the absence of BCG vaccination (Figure 3A), the IFNG test detects ~40% of infected cattle at 2 weeks post-infection compared to ~10% using the Enferplex test (no IDEXX-positives at this time point). By 6 weeks post-infection (w58), most infected cattle are detected by the Enferplex (90%) and IFNG (80%) tests, while ~40% are detected using IDEXX. Following a skin test (at w64), all infected cattle are detected by all three tests one week later (w65, 13 weeks post-infection).

In contrast, test responses in the BCG vaccinates (Figure 3B) illustrate the issue of BCG-driven PPDB-biased responses, causing ~70% false IFNG positives and ~40% Enferplex positives prior to *M. bovis* infection. The IDEXX test responses, however, remained lower in BCG vaccinates until after infection, requiring a skin test for maximum sensitivity of infection detection.

### 3.3. M. avium subsp. paratuberculosis (MAP) Antibody Test Cross-Reactivity

**Figure 4:**
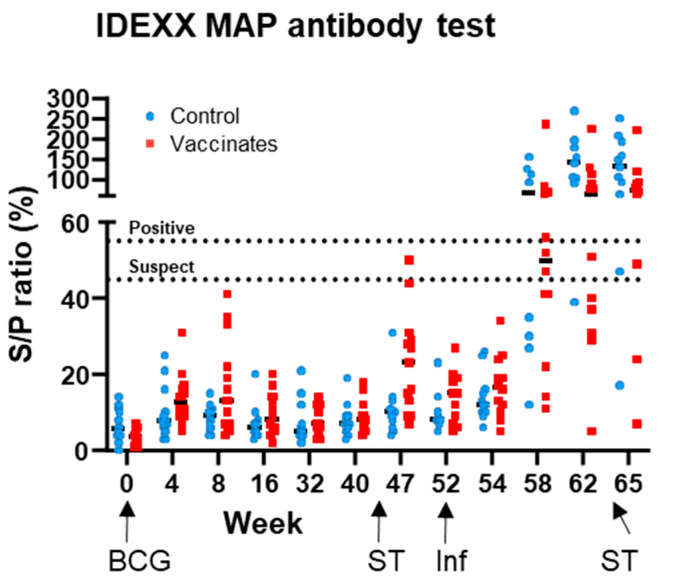
**MAP serology responses following BCG vaccination and *M. bovis* infection.** Test outcomes from the IDEXX MAP antibody test. Each symbol represents an individual animal with an assay positivity cut-off displayed with a dotted line. Unvaccinated control animals are represented in blue and BCG-vaccinated animals in red.

To assess the potential impact of BCG vaccination upon MAP diagnostic antibody testing, all experimental sera were tested using the MAP IDEXX antibody test. Figure 4 shows the MAP test responses for BCG-vaccinated and unvaccinated control cattle at each time point. The percent of test-positive individuals within the BCG-vaccinated and unvaccinated cattle is also summarised in Table 2. Pre-*M. bovis* infection, there were no test positives in any animal, but statistically significant transient increases in MAP readouts were observed in the BCG vaccinated group which were not observed in the control group, i.e., at 4–8 weeks post-BCG vaccination and later at w47–52, after the skin test (Table 2). By 6 weeks post-*M. bovis* infection (w58), MAP test positives were observed in both BCG vaccinates and controls. These responses appeared to peak by 10 weeks post-infection (w62) and were not enhanced following a skin test. The data from this experimental infection clearly show the potential for cross-reactivity of *M. bovis*-infected animals on the MAP diagnostic antibody test but also suggest that BCG vaccination alone has little impact.

## 4. Discussion

This study explored the application of two antibody tests following BCG vaccination and experimental infection of cattle with *M. bovis*. The IDEXX *M. bovis* antibody test is a more traditional, relatively simple ELISA format with a stated lower sensitivity of 64.5%, and the Enferplex multiplex ELISA test has a higher stated sensitivity of 93.9% (at the Hi Se interpretation). Both tests have comparable stated specificities (98% and 98.4%, respectively).

Although the mainstay of testing for TB in cattle in GB is based around the detection of host cell-mediated immune responses (CMI, i.e., skin and IGRA tests), antibody testing has increased over the past seven years (using the IDEXX *M. bovis* test), both as an ad hoc third-line test for skin- and IGRA-negative cattle in infected herds across England and Wales and as a “named test” for mandatory application in Wales, e.g., for inconclusive skin reactors in infected herds. Currently in GB, the Animal and Plant Health Agency are working towards a marketing authorisation application for BCG vaccination in cattle, coupled with a new skin test reagent that is able to detect infected amongst vaccinated animals (DIVA test). BCG vaccination is known to be a confounder of the comparative PPD skin and IGRA tests, but less is known about the impact upon diagnostic antibody tests—the aim of this study. The results presented herein show that the impact of BCG vaccination itself was minimal on either of the *M. bovis* diagnostic antibody tests utilised. A small number of positive responses occurred on both tests during the vaccination period, which are likely to be non-specific false positive results.

Strikingly, a pre-infection tuberculin skin test generated more pronounced test positivity in the Enferplex (Hi Se) test compared to the IDEXX *M. bovis* test. Eight of the twelve BCG-vaccinated animals were positive to the Enferplex (Hi Se) test when sampled at some timepoint between study week 42 (time of the first skin test) and study week 52 (time of *M. bovis* infection), while only three were positive to the IDEXX *M. bovis* test. The anamnestic boost of antibody responses by a prior tuberculin skin test is well documented [25]. In our current study, these responses were all transient but suggest a potential specificity issue for the Enferplex test following BCG vaccination and tuberculin skin test anamnestic boost. However, it should be highlighted that control policies involving BCG vaccination of cattle would require replacing the current tuberculin skin test with a DIVA skin test to maintain a high specificity in surveillance testing. One such candidate reagent is DST-F, a recombinant fusion protein containing the defined mycobacterial antigens ESAT6, CFP10, and Rv3615c [14]. Unfortunately, due to the confines of the current cattle experiment, we were not able to independently assess and compare the impact on serology test specificity of skin testing with either tuberculin or the DST-F reagent. The ability of the DST-F skin test alone to induce an anamnestic boost to antibody responses and the impact that this has on the sensitivity and specificity of bTB serology tests performed in infected and BCG-vaccinated animals, respectively, is an area for future study.

As previously mentioned, the cattle in our current study were skin tested with not only PPDA and PPDB (in which MPB83, and to a lesser extent MPB70, is a dominant component) but also DST-F, which altogether may have generated stronger serological responses, particularly in the multi-antigen Enferplex test, than would normally be observed. This might explain the contrast with previously published data for the Enferplex test where 39 BCG-vaccinated cattle gave no positive responses post-skin test [17]. Alternatively, this previous study was conducted using an earlier version of the Enferplex test, potentially lacking antigens that were being recognised by antibodies in the serum of animals in our current study.

Following *M. bovis* infection, IDEXX *M. bovis* test responses were initially higher in the unvaccinated control group compared to the BCG-vaccinated group, while positive Enferplex responses post-infection appeared equally in both control and vaccinated cattle. Overall, our data support the earlier detection of infection by the Enferplex test compared to IDEXX, suggested by their respective stated sensitivities, but given time and a skin test, both tests identified all of these experimentally infected cattle. The lower antibody responses in BCG vaccinates relative to controls post-*M. bovis* infection is in keeping with the significant reduction in gross pathology scores from animals within the vaccinated group previously published for these cattle [22], reflecting the modulation of immune responses by vaccination with a reduced disease burden.

This current study showed limited evidence of BCG-driven non-specific serological responses on the IDEXX MAP antibody test. Although responses to the assay did increase after BCG vaccination and pre-infection skin test in the BCG group, the resulting increase did not lead to any test-positive animals. The biggest driver in MAP positivity was infection with *M. bovis,* which produced a significant number of test-positive animals. As all were MAP test negative prior to the infection stage, the data suggest a cross-reactivity of *M. bovis*-driven antibodies on the MAP test, which uses a whole cell lysate to capture antibody rather than MAP-specific antigens. Thus, this lysate could contain mycobacterial epitopes that are shared between *M. bovis* and MAP. The additional reagent used in the skin test for this experiment cannot explain this cross-reactivity, since the DST-F antigens are not active in MAP. A previous study [21] has highlighted non-specific MAP responses in sera from another TB challenge study; however, it is worth noting that in this study these cross-reactive responses were only seen in experimentally infected animals and not those naturally infected with TB, although the number of animals tested was low. As our study only contained animals experimentally infected with *M. bovis*, further work is needed to assess whether such non-specific reactivity would occur in naturally *M. bovis*-infected animals. In support of this, Lilenbaum et al. previously demonstrated 7 out of 32 naturally infected bTB reactor animals (i.e., tested positive for SICCT and IGRA) also tested positive using a panel of MAP serology tests, whilst remaining faecal MAP culture negative [20]. Furthermore, Byrne et al. demonstrated that the MAP status of a cattle herd as defined using a serology-based diagnostic test was positively associated with a bTB episode, suggesting the potential for increased numbers of false-positive results [26]. Thus, these results highlight the potential for misdiagnosis of MAP infection when using serology tests in bTB-infected cattle.

## 5. Conclusions

In summary, we have shown that BCG vaccination per se does not significantly impact the specificity of serological diagnostic tests for bTB or Johne’s disease. However, caution should be taken when assessing results from serology tests for bTB if tuberculin skin testing has occurred shortly before blood collection from BCG-vaccinated cattle. Additionally, *M. bovis* infection gives rise to false-positive test results for Johne’s disease irrespective of the animals’ prior BCG vaccination status, although testing of serum samples from additional *M. bovis* challenge studies will be required to strengthen this observation.

## Figures and Tables

**Table 1:** Test positivity for *M. bovis* antibody assays.

	*M. bovis* IDEXX: % Positive (Number Positive/Total)		*M. bovis* Enferplex (Hi Se): % Positive (Number Positive/Total)		*M. bovis* Enferplex (Hi Sp): % Positive (Number Positive/Total)	
Week	Control Group	BCG Group	Control Group	BCG Group	Control Group	BCG Group
0	0 (0/14)	0 (0/14)	0 (0/14)	0 (0/14)	0 (0/14)	0 (0/14)
4	0 (0/14)	7 (1/14)	7 (1/14)	0 (0/14)	7 (1/14)	0 (0/14)
8	0 (0/13)	8 (1/13)	0 (0/13)	0 (0/14)	0 (0/13)	0 (0/14)
16	0 (0/13)	0 (0/13)	0 (0/13)	0 (0/13)	0 (0/13)	0 (0/13)
32	0 (0/13)	0 (0/13)	0 (0/13)	0 (0/13)	0 (0/13)	0 (0/13)
40	0 (0/13)	0 (0/13)	8 (1/12)	0 (0/13)	0 (0/12)	0 (0/13)
47	0(0/13)	23 (3/13)	15 (2/13)	54 (7/13)	8 (1/13)	38 (5/13)
52	0 (0/12)	0 (0/11)	17 (2/12)	42 (5/12)	8 (1/12)	25 (3/12)
54	0 (0/11)	0 (0/12)	9 (1/11)	25 (3/12)	0 (0/11)	0 (0/12)
58	38 (3/8)	0 (0/12)	90 (9/10)	100 (12/12)	70 (7/10)	75 (9/12)
62	67 (6/9)	8 (1/12)	100 (9/9)	100 (12/12)	100 (9/9)	100 (12/12)
65	100 (11/11)	100 (11/11)	100 (11/11)	100 (11/11)	100 (11/11)	100 (11/11)

**Table 2:** IDEXX MAP antibody test results.

	Median S/P Ratio (%) [95% CI of the Median]		Test Outcome % Positive (Number Positive/Total Tested)	
Week	Control Group	BCG Group	Control Group	BCG Group
0	5.5 [4, 11]	3.5 [2, 6]	0 (0/14)	0 (0/14)
4	7.5 [4, 16]	12.5 [9, 17] *	0 (0/14)	0 (0/14)
8	9 [5, 11]	13 [6, 33] **	0 (0/13)	0 (0/14)
16	6 [4, 8]	8 [5, 14]	0 (0/13)	0 (0/14)
32	5 [4, 12]	7 [4, 12]	0 (0/13)	0 (0/13)
40	7 [5, 9]	8 [5, 12]	0 (0/13)	0 (0/13)
47	10 [5, 13]	23 [9, 31] **	0 (0/13)	0 (0/13)
52	8 [8, 14]	15 [6, 20] *	0 (0/12)	0 (0/11)
54	12 [9, 25] *	16.5 [10, 24] ***	0 (0/11)	0 (0/12)
58	64.5 [12, 157] ***	49.5 [22, 69] ****	50 (4/8)	42 (5/12)
62	141 [92, 197] ****	64 [31, 114] ****	89 (8/9)	50 (6/12)
65	131 [47, 208] ****	71 [24, 120] ****	82 (9/11)	64 (7/11)

* *p* < 0.05, ** *p* < 0.01, *** *p* < 0.001, **** *p* < 0.0001, Kruskal–Wallis test with Dunn’s multiple comparisons test (compared to week 0 values).

## Data Availability

The datasets used and/or analysed during the current study are available from the corresponding author on reasonable request.
